# Computational Prediction and Experimental Values of Mechanical Properties of Carbon Nanotube Reinforced Cement

**DOI:** 10.3390/nano11112997

**Published:** 2021-11-08

**Authors:** Carlos Talayero, Omar Aït-Salem, Pedro Gallego, Alicia Páez-Pavón, Rosario G. Merodio-Perea, Isabel Lado-Touriño

**Affiliations:** 1 School of Architecture, Engineering and Design, Universidad Europea de Madrid, 28670 Madrid, Spain; alicia.paez@universidadeuropea.es (A.P.-P.); rosario.gomezdemerodio@universidadeuropea.es (R.G.M.-P.); misabel.lado@universidadeuropea.es (I.L.-T.); 2 Hexagon HMI, 28050 Madrid, Spain; omar.ait-salemduque@hexagon.com (O.A.-S.); pedro.gallego.garcia@alumnos.upm.es (P.G.)

**Keywords:** computer model, carbon nanotubes, mechanical properties, Young’s modulus, microstructure, reinforced cement

## Abstract

The main objective of this study is to create a rigorous computer model of carbon nanotube composites to predict their mechanical properties before they are manufactured and to reduce the number of physical tests. A detailed comparison between experimental and computational results of a cement-based composite is made to match data and find the most significant parameters. It is also shown how the properties of the nanotubes (Young’s modulus, aspect ratio, quantity, directionality, clustering) and the cement (Young’s modulus) affect the composite properties. This paper tries to focus on the problem of modeling carbon nanotube composites computationally, and further study proposals are given.

## 1. Introduction

From new materials in construction to medical research, the use of carbon nanotube (CNT) composites has rapidly increased such that they are considered one of the materials of the future. Their excellent mechanical properties and the low quantity of them required to obtain significantly improved composite properties make CNT an ideal inclusion in the nanotechnology industry.

Carbon nanotubes are graphene sheets arranged in cylindrical shapes. This hollow tube consists of carbon hexagons and may be capped by two spherical caps at both ends [1,2]. There are two types of CNT, depending on the number of internal layers they have: single-walled carbon nanotubes (SWCNT) and multi-walled carbon nanotubes (MWCNT). SWCNTs consist of a single-layer tube with a diameter in the range of nanometers, while MWCNTs consist of two or more concentric layers with different diameters (no more than ten nanometers of outer diameter).

The CNT atomic structure can be defined by the tube chirality or helicity, described by its chiral vector and chiral angle. The chiral angle is an indicator of the twisting of the tube. These nanoparticles show excellent electrical and mechanical properties and chemical stability. In this sense, the addition of small concentrations of CNT to cement paste enhances cement performance, since it increases the mechanical and electrical performance and the durability of the material [3]. These novel composites expand their field of application from conventional construction to communication, medicine, and space. These new cements can be used in applications such as sensors to monitor damage of structures, to control the corrosion of metallic materials in reinforced concrete, for heating mechanisms, as cathodic protection systems, for the protection of electrical systems and components sensitive to electromagnetic radiation, as antistatic floors for the electronic instrumentation industry and hospital floors, etc. [4,5,6,7,8].

Over the past years, several (both theoretical and experimental) studies about the addition of CNT to building materials, such as cement, have been developed to obtain accurate results about the properties of this material [9,10,11].

Current studies show that the addition of carbon nanoparticles to cement paste provides a significant increase in their mechanical properties and durability. However, chemical interactions between nanoparticles and cement cause modifications in the hydration process of the material and in the formation of Ca(OH)_2_, which leads to microstructural changes and variations in the final properties of the material [12,13]. Despite the great interest that this topic has been generating recently, there is still little information about how these nanostructures affect the hydration processes of the material, its rheological properties, its hardening mechanisms, etc. [13,14,15,16]. Moreover, conclusions and values vary too much amongst all investigations [17,18]. These discrepancies make it even more difficult to build a solid model to predict the real behavior of composites with these kinds of inclusions.

Therefore, systematic studies are still needed about the effects of the addition of different types of nanoparticles on the properties of cement paste. Due to the high price of these materials, approaching their study by simulation methods can be a useful alternative for carrying out these systematic studies. Simulations and virtual modeling play an important role in understanding the response of these types of materials.

The main goal of this paper is to create a solid modeling process to simulate virtual testing of CNT composites in order to optimize laboratory work. Semi-analytical models and the finite element method were used to develop the simulations.

## 2. Materials and Methods

This research followed three steps: virtual model according to previous research and manufacturing properties, physical testing, and correlation and model adjustment.

For the first step, the CNT manufacturer data sheet (Sigma-Aldrich^®^, Taufkirchen, Germany) was considered. In addition to that, previous testing data [19] was useful for establishing the first adjustment of the computing model.

### 2.1. Materials

Table 1 shows the properties of the different types of CNT and the cement used during the study. The diameter, length, and density of the CNT were taken from the material supplier datasheet. The Young’s modulus comes from a variety of sources that agree on the value of this property [19].

During the experimental phase, three types of composite were developed. The additions to the cement were as follows: 0.02 wt.% of SWCNT; 0.1 wt.% of MWCNT; 0.02 wt.% of SWCNT + 0.2 wt.% of the dispersant sodium dodecyl sulfate (SDS). Control cement samples were also evaluated. The composition of each kind of composite is described in Table 2.

### 2.2. Methods

#### 2.2.1. Experimental Procedure

All of the steps and considerations are valid for the preparation of all types of composites. First of all, cement paste has to be prepared for all samples. The weight ratio of cement to water is 2:1. Then, the next steps are as follows:

1. Dispersion of the CNT in water. CNTs are mixed with water. Then, the mixture is ultrasonicated in a horn sonicator (Sonoplus HD 2070, Bandelin, Berlin, Germany) for 1 h.
2. Mixture and preparation of samples. The mixture, CNT and water, is added to cement paste. This procedure is done according to the standard UNE EN 1961:2005. With the cement paste, cylindrical specimens of 2 cm height and 1 cm diameter are molded for the mechanical properties’ measurements.
3. Testing. After 28 days, composite samples can be tested. Firstly, specimens are measured. The samples are cylindrical, 20 mm in the longitudinal direction and 14.3 mm in diameter. Two trials were developed: compressive strength test, according to UNE EN 12390-3, and indirect tensile test, according to UNE EN 12390-6. Clamping jaw displacement speed for the compressive and indirect tensile tests is 77 N/s and 24 N/s, respectively. Five samples are tested for each case.

**Figure 1:**
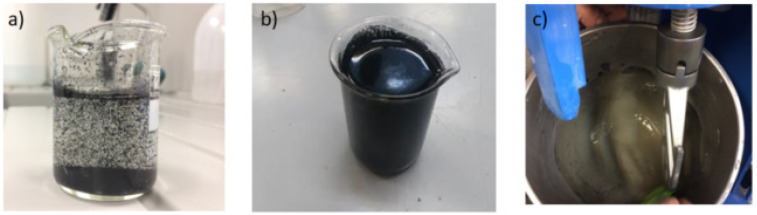
(**a**) Water + carbon nanotubes before ultrasonic dispersion; (**b**) water + carbon nanotubes after ultrasonic dispersion; (**c**) water + carbon nanotubes dispersion + cement paste after mixing.

**Figure 2:**
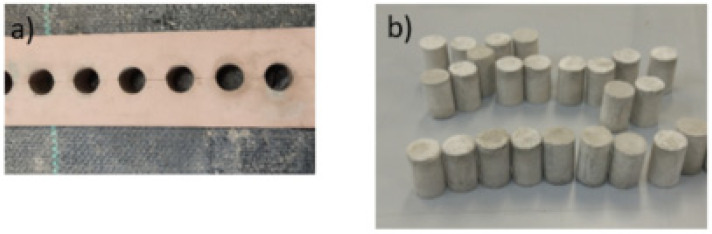
(**a**) Molds used to obtain specimens; (**b**) cylindrical specimens.

Figure 1 and Figure 2 show the different stages of the experimental procedure, from the dispersion of the nanotubes in water, through obtaining the reinforced cement paste, to the molding of cylindrical testing samples for mechanical properties.

**Figure 3:**
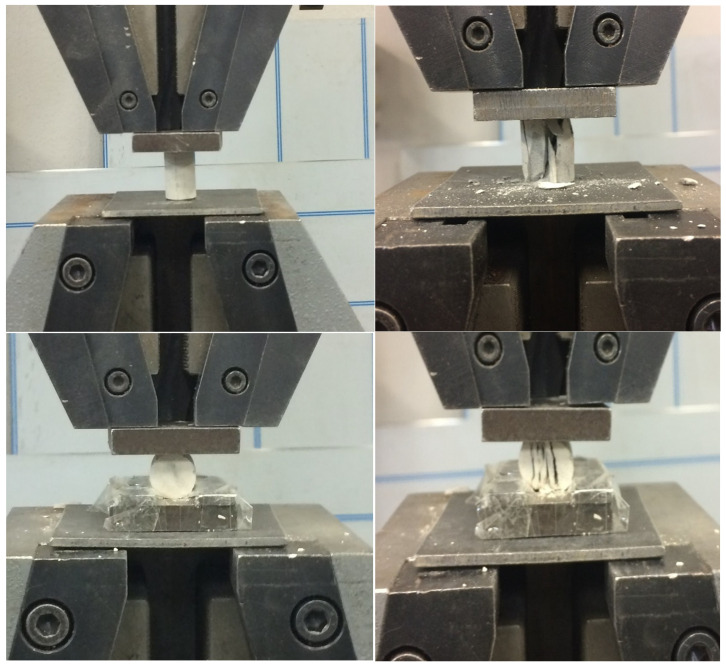
(**upper**) Compressive strength test; (**lower**) indirect tensile strength test.

Figure 3 shows the tests developed. To perform the mechanical properties tests, the samples were placed between two steel plates that provided a stiffness high enough relative to the cement limits to ensure that pressure was exerted homogeneously over the surface of the sample.

#### 2.2.2. Computational Model

For the creation of the computational model, the software Digimat (version 2021.1 MSC Software, Irvine, CA, USA) [20] was chosen and the modules MF (Mean-Field) and FE (Finite Element) were used. Digimat MF is based on a semi-analytical model, while Digimat FE is based on the finite element method.

#### From Micro- to Macroscopic Scale: RVE Concept

Material complex modeling is firstly done on a microscopic scale to make sure that all phases and parameters are well configured. However, solving a mechanical problem in a microscopic way is not a good approach to the macroscopic behavior of materials. For this reason, there are two distinguished scales: microscopic and macroscopic [21,22].

The way Digimat links these two scales is using the representative volume element (RVE) concept. The idea of this volume is that it acts as a transition element between both scales.

Thus, RVE must be sufficiently large to represent macroscopic scales and small, with respect to the size of the solid body, to represent microscopic scales.

With this, each point in the solid (macroscopic scale) is the center of a representative volume element (RVE). Furthermore, RVE is composed of a finite number of inclusions to represent the microscopic scale [23]. 

Understanding the RVE concept can help in choosing the right parameters to obtain a good representative volume. The size of the RVE can either be automatically computed by Digimat FE, based on the microstructure definition of the composite, or manually. The software automatically computes an adapted size for the RVE taking into account the size of inclusions [24].

#### CNT Dispersion

Using Digimat Software, we can choose and change the CNT distribution. The orientation of each inclusion is defined by a unit vector $\bar{p}$ along its axis of revolution. Then, it is placed in 3D space with two spherical angles: *θ* and *φ*.

The direction $\bar{p}$ varies amongst the inclusions. Thereby, an orientation distribution function (ODF) $\psi (\bar{p})$ is defined, where $\psi (\bar{p})d(\bar{p})$ is the probability of finding inclusions in the solid angles ($\bar{p},\;\bar{p}+d\bar{p}$).

The composite material obeys this condition:

(1) $$\nu_{0}+\overset{N}{\underset{i=1}{\sum }}\nu_{i}=1$$

where:

$\nu_{0}$ is the volume fraction of the matrix phase;

$\nu_{i}$ is the volume fraction of each family of inclusion in the composite;

i is each family of inclusion in the composite.

Inclusions obey these conditions:

(2) $$\psi_{i}(\bar{p})=\psi_{i}(-\bar{p})$$

(3) $$\oint \psi_{i}(\bar{p})d(\bar{p})=1$$

The first expression means that two inclusions with opposite directions are the same inclusion. The second equality is a normalization of the probabilities: the sum of probabilities equals 1.

**Figure 4:**
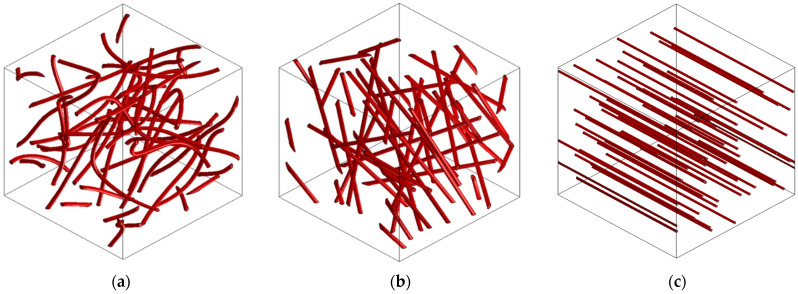
(**a**) RVE of realistic CNT composite; (**b**) RVE of a simplified CNT composite; (**c**) RVE with aligned inclusions.

Fiber distribution inside the matrix is a huge issue in CNT composites, since fabrication is not easy. For that reason, when we want to solve the problem in a computational way, different kinds of models must be taken into account. Figure 4a shows how CNTs (with the right fabrication process or with dispersant added to ensure homogenization) are placed in a volume. However, to make an analysis of this RVE, we need to mesh it, and, using this model, this would be very difficult, with a high computational cost due to CNT curvature. Thus, Figure 4b shows a simplified model with which we obtain reliable results. Figure 4c refers to a model in which the inclusions are aligned to the load applied. This model will be considered and explained later.

Two element types were used to create the mesh: voxel and tetrahedral. Both offer similar results if the element size is well configured. CNTs are inclusions with a very high aspect ratio. For this reason, it is important to make a fine mesh. However, computer memory is a limiting factor. Using voxel elements for our simulations, each direction is divided into 120 parts; that is to say, the RVE is meshed with 1,728,000 elements. If tetrahedral elements are used, the default element size is considered.

## 3. Results

**Figure 5:**
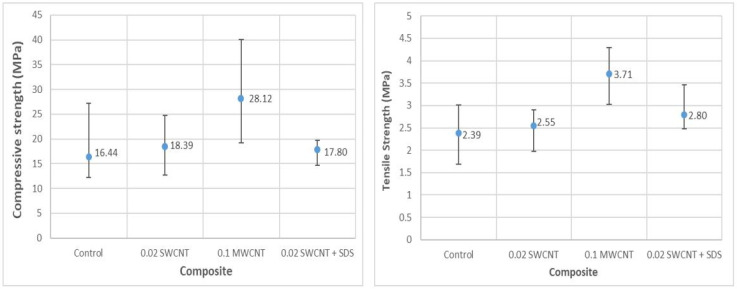
(**left**) Compressive strength average value with deviation bars; (**right**) indirect tensile strength average value with deviation bars for control cement and each type of composite.

The following plot (Figure 5) and Table 3 show the average and extreme values obtained in the compressive strength and tensile strength laboratory tests for each type of CNT composite. Deviation bars are included to reflect how the data are scattered (five tests for each case except for 0.02 SWCNT + SDS, where only three tests were valid).

The above data correspond to all experimental laboratory tests. As can be seen, the addition of CNTs to the cement paste increased the mechanical properties of the final material, as reported in the bibliography [3,6].

The addition of dispersants to reinforced cement paste is important to avoid the formation of agglomerates of the nanomaterials and to ensure a good dispersion of these in the cement paste [25]. As can be seen in the experimental results, when adding the SDS dispersant, the value of the standard deviation decreased, which implies that the results were more consistent and, therefore, the dispersion of the nanotubes in the cement paste was improved.

However, one of the most important parameters in the field of materials is Young’s modulus, since it provides a measure of the stiffness of the material and it can be related to its strength. Young’s modulus has been calculated according to EHE-08 [26], which is the Spanish regulation that establishes the requirements and calculation procedures related to structural concrete. The reason for using this expression is that for fragile materials it is more robust than the calculation derived from experimental plots. The relation is defined by:

(4) $$Y=8700\times \sqrt[{3}]{Rc}$$

where:

Y is the Young’s modulus;

Rc is the compressive strength of the materials.

**Figure 6:**
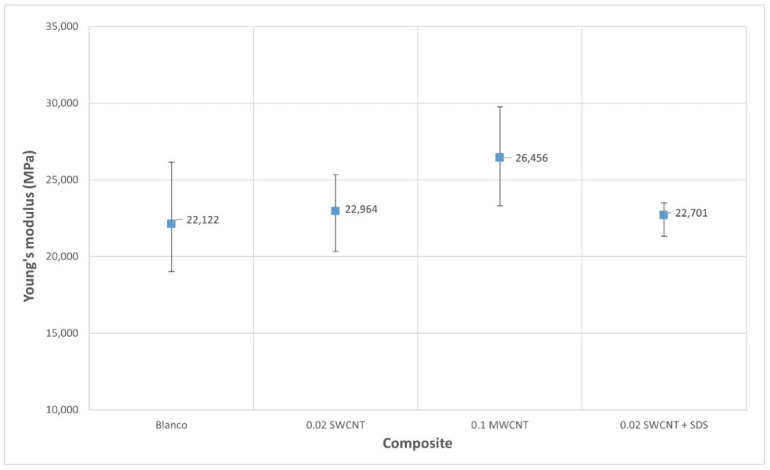
Young’s modulus for each type of composite calculated with (4). Average values and deviation bars.

With this, the Young’s modulus for each type of composite is calculated, as shown in Figure 6 and Table 4, where mean values and deviation are represented:

Once experimental results have been presented, the simulations have to be developed. In the following table (Table 5) are shown all the experimental results obtained, the relative deviation of each measure with respect to the mean value, the computational value for each type of composite, and the experimental error between the computational value and each experimental result obtained in the laboratory for each case. It is important to highlight that the computational model only offers one value. The mean value is calculated and shown with no deviation. As was mentioned before, Digimat models have been developed for a good CNT distribution inside the matrix. The dispersant is included to achieve good homogenization. For this reason, the computational model is a good representation of this composite. However, as it is not possible to predict the orientation of the composite with no dispersant added (0.02 SWCNT), the computational model for 0.02 SWCNT and 0.02 SWCNT + SDS is the same. In the last section of this article, the influence of CNT distribution is evaluated.

## 4. Discussion

With the results shown in Table 5, we can see that computational model results are closer to the 0.02 SWCNT + SDS composite than to 0.02 SWCNT. That is to say, the computational model is a good approximation to well-dispersed inclusions models.

Although the experimental error for the 0.02 SWCNT composite is in an appropriate range, the mean value of 0.1 MWCNT is too high. Using Digimat MX, both curves, experimental and computational, can be adjusted to obtain values of Young’s modulus that allow us to reduce the experimental error. It is important to note that the Young’s modulus of the CNT is taken from different sources [11,14], and, therefore, its influence should be evaluated. Furthermore, the Young’s modulus of cement, as shown in Table 4, has a deviation between its maximum and mean value and between its minimum and mean value of 18% and 14%, respectively. For this reason, the Young’s moduli of both the CNT and of cement are variables that need to be studied.

After performing different virtual tests with Digimat MX, the results show that changing the values of the basic properties of the CNT described in Table 1 does not substantially change the characteristics of the composites. The evidence of this is shown in Table 6. In a range between 1.1 and 4 TPa for the Young’s modulus of CNT, we observed an improvement of only 2% in the experimental error. Besides, it would be necessary to have a CNT with a Young’s modulus of 18 TPa to obtain 0% experimental error with respect to the mean value obtained in the laboratory tests, which is not a realistic value. All of this information informed us that if we want to reduce the experimental error, we need to focus on cement properties.

On the other hand, by using the values of all of the measures of the control cement Young’s modulus made in the laboratory, it is possible to make a simulation to study the influence of the cement in the composite properties and to obtain the error with respect to the experimental values for each case. This is shown in Table 7, where the cement Young’s modulus is obtained from the tests and a constant value for the CNT Young’s modulus is taken. The mean value, shaded in the table, is not a direct measure.

Considering these results, it is noteworthy that if we want to reduce the experimental error between the mean value of the composite Young’s modulus and the virtual value of the 0.1 MWCNT composite, we need to consider a greater value of the cement Young’s modulus than the value that had initially been considered. If we take the maximum value of the control cement Young’s modulus obtained in the laboratory, we would achieve 0% experimental error. However, the rest of the values for the other composites would worsen. For this reason, a compromise solution needs to be found.

Finally, 23,300 MPa was chosen as the Young’s modulus of the control cement, since the computational 0.1 MWCNT result is within the experimental range, and the results for the other composites do not significantly worsen. With this, the improvement of the experimental error for the 0.1 MWCNT composite and the new data for the 0.02 SWCNT composites are shown in Table 8.

As can be seen, for the 0.1 MWCNT composite the mean value experimental error decreased by 4%, the maximum by 4%, and the minimum by 3%.

The experimental error for the mean value also decreased in the 0.02 SWCNT composite by 1%. The maximum value experimental error decreased by 4%, and the minimum increased by 7%. Although the experimental error for the minimum value increased, the results for the 0.02 SWCNT composite also improved with the change of the control cement Young’s modulus.

As mentioned earlier, during the experimental phase, some CNT aligned areas were found when breaking MWCNT specimens. The inclusions were arranged as threads aligned with the load applied, and this is the reason why we created the third model shown in Figure 4c.

**Figure 7:**
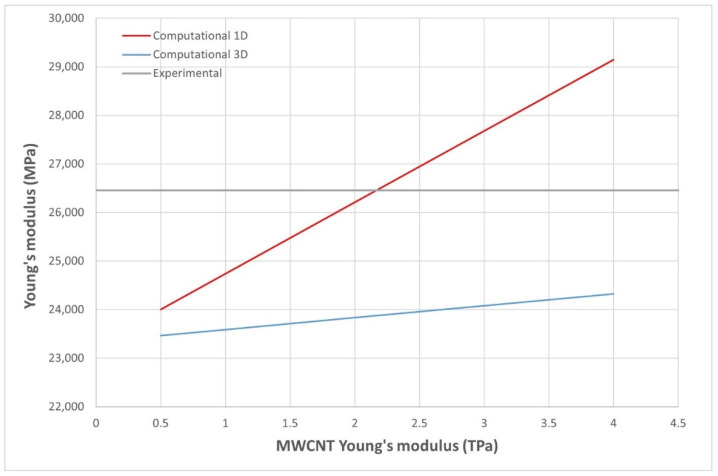
Young’s modulus for the 0.1 MWCNT composite, varying the MWCNT Young’s modulus in the range of the experimental measured values (shown in Table 4).

With this, a comparison between both models and experimental results (Table 4) is made for the 0.1 MWCNT composite in Figure 7, where the Young’s modulus of the MWCNT is varied to achieve the measured composite Young’s modulus. “Computational 3D” refers to the model in Figure 4b, and “computational 1D” refers to the model in Figure 4c. As can be seen, computational 1D results are coherent with the experimental values when aligned fibers are found, including the maximum experimental value (Table 4). The reason for this defect when SDS is not added to MWCNT composites could be the larger size of those CNTs (Table 1) compared to the SWCNT, where no orientation problems were detected even when higher concentrations were tested. It is notable that the 1D model is created only for studying an undesirable behavior (i.e., the orientation is not controlled during the manufacturing process), the 3D model being the reference for well dispersed CNTs.

### Sensitivity Analysis

The last section of this article is about determining the sensitivity of composite Young’s moduli to the most relevant parameters. Those variables are the number of CNTs inside the matrix, % weight (NT%), the aspect ratio of these (AR), the Young’s modulus of cement (E_C) and CNT (E_NT), the percentage of clustering areas inside the composite, and the directionality of the CNTs (DirX A and DirY).

For the measurement of the influence of each parameter, a machine learning software called Odyssee (version 2021.3 ODYSSEE A-Eye, MSC Software, Irvine, CA, USA) [27] was used.

The process, using machine learning based on reduced order modeling, starts with introducing the input data, simulation results in our case. The system learns from the input data, searching for patterns. There are different kinds of machine learning algorithms. Then, the system predicts the responses for other cases, and, finally, this prediction is validated with experimental results. Thus, we need to make some simulations to train the model and others to validate it.

Sixty-nine Digimat simulations were developed, varying all the parameters mentioned above; 55 of them were used to train the model and the rest to validate it. For this case, Odyssee computational speed was less than 1 s.

The solver used by Odyssee is composed of a decomposition method and an interpolation method. In this case, the solver that best suits the validation cases uses POD (proper orthogonal decomposition) for decomposition and Kriging-(linear, h3) (also known as the regression method in Gaussian processes) for interpolation. This interpolation method is a geostatistical interpolation method for predicting points. It is based on the fact that the variation of properties follows a homogeneous pattern; that is, if at a point p a certain parameter is x, then it will be more likely to find values of the variable near x the closer we are to point p. However, at a point away from p, we will not find values of the variable near x.

The goal is to interpolate the value $Z\left(x_{0}\right)$ of a random field Z(x) at the position $x_{0}$ where values are not known. $Z\left(x_{0}\right)$ is calculated from the values of the field $Z\left(x_{i}\right)$ at nearby points (*i* = 1 … *n*) where their values are known.

The solver calculates an estimator $\hat{Z}\;\left(x_{0}\right)$ of $Z\left(x_{0}\right)$ using the following expression:

(5) $$\hat{Z}\;\left(x_{0}\right)=\overset{n}{\underset{i=1}{\sum }}w_{i}\left(x_{0}\right)\;Z\left(x_{i}\right)$$

where $w_{i}\left(x_{0}\right)$ is the *i*-point weight.

The above expression is a linear combination for the calculation of the Kriging estimator so that the variance is minimal. In our problem, the field Z(x) is the Young’s modulus of composites (*E*).

**Figure 8:**
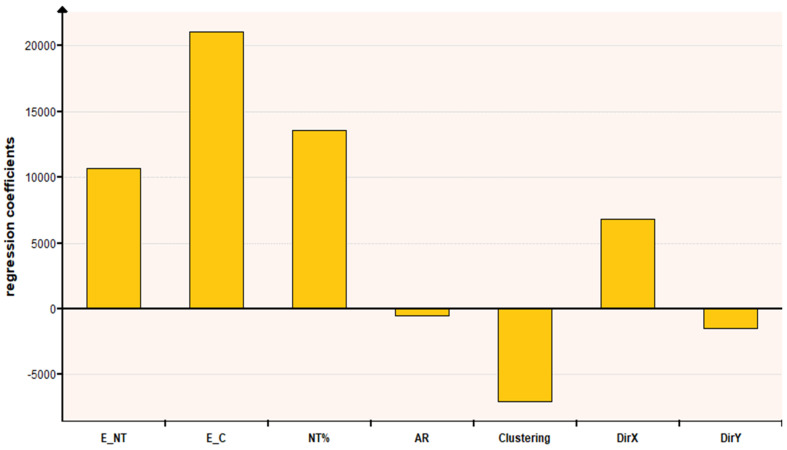
Regression coefficient of each parameter.

Figure 8 shows the regression coefficient of each parameter, that is to say, a measure of the global influence of each variable on the Young’s modulus of composites. In the diagram, it is clear that, as we mentioned before, the Young’s modulus of the cement is the parameter with the greatest impact. Then, the number of CNTs and the Young’s modulus of them are important too. The aspect ratio of the CNTs is not significant.

The variation of the parameters was chosen according to common and fair values and to experimental results obtained.

**Figure 9:**
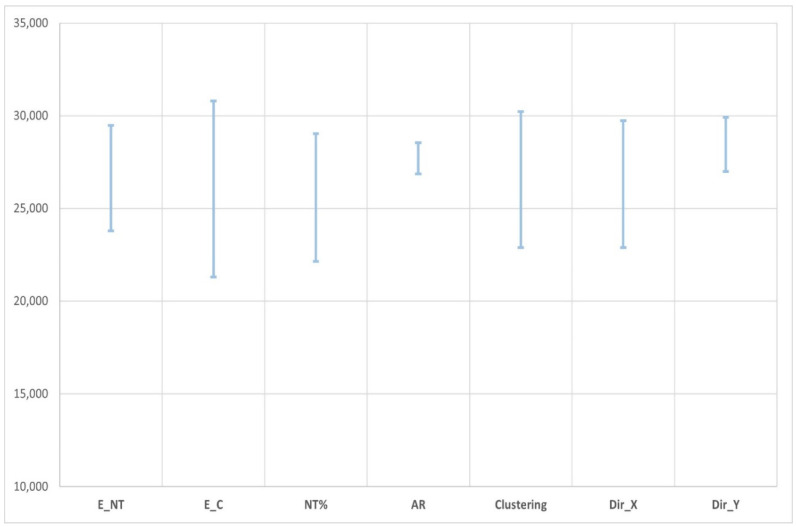
Impact of each parameter while the others are fixed for the nominal case.

From the machine learning model created, it is possible to select any case (any combination of parameters) that is inside the range of the variables and see the influence of them for that case. It is also possible to see what happens when all parameters are fixed except one, which varies within its range. Figure 9 shows the impact of each parameter (while the others are fixed) for the nominal case detailed in Table 9.

In the previous figure, it is shown what happened when, for a case selected, other parameters’ values were considered. It can be very useful to adjust the model when a case is being evaluated.

## 5. Conclusions

Using CNTs’ and cement’s basic mechanical properties, it was possible to develop a computational model that approaches experimental results with around 10% experimental error, this error being less than the deviation of the experimental results. Furthermore, computational results can be obtained in a matter of seconds when Digimat MF is used with a DELL Precision M4800 Intel (R) Core(TM) i7-7820HQ CPU @ 2.90 GHz computer or in minutes or hours when Digimat FE is used with the same computer, while experimental results cannot be obtained before 28 days (cement curing time). This is one of the keys and advantages of computational material testing. Another advantage is the cost of the computational resources vs. the cost of CNTs and facilities.

The computational 3D model works worse than the computational 1D model for composites with no dispersant added and a higher percentage of CNT composite, due to defects in the samples’ manufacture. However, the computational 3D model works better for models with dispersant added to guarantee mixture homogeneity. This shows that modeling CNT composites is very difficult and all parameters must be considered.

For this reason, a machine learning model of this material was created, varying parameters in the right range. The Young’s modulus of the cement is the parameter with the greatest impact; that is to say, it is very important to manufacture specimens following all of the steps to reduce the deviation in the measurements. With this, it is possible to evaluate each case (each parameter combination desired) easily to understand how variables affect that case.

In conclusion, for the analysis of the material, three models were developed: one in Digimat MF, one in Digimat FE, and, finally, another in Lunar, based on the analysis of Digimat.

## Figures and Tables

**Table 1:** Properties of the CNT used.

Material	Diameter (nm)	Length (μm)	AR ^1^	$\mathrm{Density}\;(g/{\mathrm{cm}}^{3})$	Young’s Modulus (MPa)	Poisson’s Ratio
Cement II/B-L 32.5 R	-	-	-	3.1	19,057	0.22
SWCNT	0.78	1	1282.05	1.8	971,000	0.28
MWCNT	12	10	833.33	2.1	1,100,000	0.30

^1^ AR: aspect ratio = length/diameter.

**Table 2:** Information about each composite.

Composite	Nanoparticles (wt.%)	Information
Control	0	Cement sample, not reinforced
0.02 SWCNT	0.02	Single-wall carbon nanotubes
0.1 MWCNT	0.1	Multi-walled carbon nanotubes
0.02 SWCNT + SDS	0.02	Single-walled carbon nanotubes + 0.2% SDS (dispersant)

**Table 3:** Compressive strength and indirect tensile strength test results for each type of composite.

	Control	Dev (%)	0.02 SWCNT	Dev (%)	0.1 MWCNT	Dev (%)	0.02 SWCNT + SDS	Dev (%)
**Mean comp. strength (MPa)**	16.44	-	18.39	-	28.12	-	17.80	-
**Max. comp. strength (MPa)**	27.2	65	24.73	34	40.02	42	19.72	11
**Min. comp. strength (MPa)**	12.24	25	12.75	31	19.24	32	14.72	17
**Mean indirect tensile strength (MPa)**	2.39	-	2.55	-	3.71	-	2.80	-
**Max. indirect tensile strength (MPa)**	3.01	26	2.90	14	4.29	16	3.47	24
**Min. indirect tensile strength (MPa)**	1.68	30	1.97	23	3.02	19	2.47	12

**Table 4:** Young’s modulus for each type of composite calculated with (4). Mean, maximum, and minimum values obtained from laboratory tests. Deviation calculated in % with respect to the mean value.

	Control	Dev (%)	0.02 SWCNT	Dev (%)	0.1 MWCNT	Dev (%)	0.02 SWCNT + SDS	Dev (%)
**Mean Young’s modulus (MPa)**	22,122	-	22,964	-	26,456	-	22,701	-
**Max. Young’s modulus (MPa)**	26,164	18	25,348	10	29,758	12	23,503	4
**Min. Young´s modulus (MPa)**	16,024	−14	20,325	11	23,312	12	21,321	6

**Table 5:** Experimental Young’s modulus values (MPa) obtained with (4) for each specimen and mean value (calculated, not measured), deviation percentage with respect to the mean value, computational model Young’s modulus (MPa), and experimental error for each composite.

**Experimental Values 0.02 SWCNT**	**Dev (%)**	**Computational Value**	**Exp. Error (%)**
25,348	10	22,187	12
24,513	7		9
22,964	-		3
22,799	−1		3
21,018	−8		−6
20,325	−11		−9
**Experimental Values 0.1 MWCNT**	**Dev (%)**	**Computational Value**	**Exp. Error (%)**
29,758	12	22,432	25
27,144	3		17
26,456	-		15
26,046	−2		14
25,142	−5		11
23,312	−12		4
**Experimental Values 0.02 SWCNT + SDS**	**Dev (%)**	**Computational Value**	**Exp. Error (%)**
23,503	4	22,187	6
23,160	2		4
22,701	-		2
21,321	−6		−4

**Table 6:** Computational results for composite Young’s modulus variation considering different CNT Young’s modulus values for a given cement Young’s modulus.

CNT Young’s Modulus (TPa)	Cement Young’s Modulus (MPa)	Composite Computational Young’s Modulus (MPa)	Error Relative to Experimental Values (%)
1.1	22,122	22,432	15
2	22,122	22,654	14
3	22,122	22,900	13
4	22,122	23,145	13
…	…	…	…
18	22,122	26,524	0

**Table 7:** Computational results for composite Young’s modulus variation considering different cement Young’s modulus values for a given CNT Young’s modulus.

Cement Young’s Modulus (MPa)	CNT Young’s Modulus (TPa)	Composite Computational Young’s Modulus (MPa)	Mean Value Experimental Error (%)
26,164	1.1	26,481	0
23,459	1.1	23,772	10
22,122	1.1	22,432	15
20,300	1.1	20,607	22
20,049	1.1	20,356	23
19,023	1.1	19,328	27

**Table 8:** Computational results of calculated CNT and composite Young’s modulus values after taking 23,300 MPa as the cement Young’s modulus.

Composite	0.1 MWCNT	0.02 SWCNT	0.02 SWCNT + SDS
Cement Young’s modulus (MPa)	23,300	23,300	23,300
CNT Young’s modulus (TPa)	1.1	0.971	0.971
Composite computational Young’s modulus (MPa)	23,612	23,365	23,365
Maximum experimental error %	21	8	1
Mean value experimental error %	11	−2	−3
Minimum experimental error %	−1	−15	−10

**Table 9:** Nominal case chosen.

E_NT (MPa)	E_C (MPa)	NT%	AR	Clustering %	Dir_X %	Dir_Y %
2,500,000	21,000	1.4	500	40	35	35

## Data Availability

The data presented in this study are available on request from the corresponding author.
